# Chiral discrimination by recollision enhanced femtosecond laser mass spectrometry

**DOI:** 10.1038/s41598-020-71069-9

**Published:** 2020-08-21

**Authors:** Jean-Luc Bégin, Maye Alsaawy, Ravi Bhardwaj

**Affiliations:** Department of Physics, University of Ottawa, Ottawa, ON K1N 6N5 Canada

**Keywords:** Circular dichroism, Near-infrared spectroscopy, Ultrafast photonics, Atomic and molecular collision processes, Atomic and molecular interactions with photons

## Abstract

Chiral molecules and their interactions are critical in a variety of chemical and biological processes. Circular dichroism (CD) is the most widely used optical technique to study chirality, often performed in a solution phase. However, CD has low-efficiency on the order of 0.01–1$\%$. Therefore, there is a growing need to develop high-efficiency chiroptical techniques, especially in gas-phase, to gain background-free in-depth insight into chiral interactions. By using mass spectrometry and strong-field ionization of limonene with elliptically polarized light, we demonstrate an efficient chiral discrimination method that produces a chiral signal of one to two orders of magnitude higher than the conventional CD. The chiral response exhibits a strong dependence on wavelength in the range of 1,300–2,400 nm, where the relative abundance of the ion yields alternates between the two enantiomers. The origin of enhanced enantio-sensitivity in intense laser fields is attributed to two mechanisms that rely on the recollision dynamics in a chiral system: (1) the excited ionic state dynamics mediated either by the laser field or by the recollision process, and (2) non-dipole effects that alter the electron’s trajectories. Our results can serve as a benchmark for testing and developing theoretical tools involving non-dipole effects in strong-field ionization of molecules.

## Introduction

Chiral molecules lack $S_n$ symmetry (improper rotation axis) due to the presence of a chiral center in which an atom is connected to four different groups of atoms^1^. Consequently, there is a handedness to the molecule. The left- and right-handed molecules are non-superimposable mirror images of each other, called enantiomers. They have identical physical and chemical properties making it difficult to differentiate them. Chiral discrimination requires an interaction between two chiral systems—a chiral reagent with known handedness that can induce enantio-selectivity in the second chiral system.

In nature, bio-molecules such as amino acids that make up life on earth are homochiral, where one enantiomer exists predominantly over the other due to asymmetric interactions^2^. Homochirality plays a pivotal role in our daily life by serving as a chiral reagent for enantio-selectivity. For example, olfactory receptors in our nose, responsible for the sense of smell, interact differently with the two enantiomers of a chiral system. For instance, the right-handed enantiomer of limonene has an orange odour while the left-handed enantiomer has a lemon odour^3^.

In practice, enantio-selectivity can be achieved using circularly polarized light (CPL) as a chiral reagent. CD is one of the most widely used chiroptical techniques. It arises due to the coupling of the electric and magnetic dipole transitions, thereby resulting in a differential absorption of left- and right-circularly polarized light^1,4–8^. CD has low-efficiency on the order of 0.01–1$\%$ because magnetic dipole transitions are involved^9–12^. Moreover, CD measurements are mostly conducted in solution phase, making it difficult to isolate the optical response of the chiral molecule from the influence of solvent-solute interactions^13,14^. Complementary techniques, such as vibrational circular dichroism and Raman optical activity^15^, have been used to specifically target the molecules in liquid media by probing the fundamental vibrational bands. However, their use in gas-phase studies has been limited. So, there is a continued effort to develop new gas-phase techniques to enhance chiral sensitivity; cavity ring-down polarimetry is one such example^16^.

Most gas-phase CD experiments employ highly sensitive techniques based on multiphoton ionization of chiral molecules instead of conventional absorption of CPL. Electrons or ions resulting from the light-matter interaction are detected using either imaging techniques such as photoelectron angular distribution^17^, mass spectrometry^18–21^ or a combination of both^22^. CD measurement with laser ionization mass spectrometry often use Resonance Enhanced Multiphoton Ionization (REMPI)^18,23–27^. They rely on resonant electronic absorption of one photon, which gives rise to the CD effect and is specific to the molecule, followed by ionization with additional photons of either the same or different colours. There have been very few studies on non-resonant laser ionization mass spectrometry^21,28^. Since the underlying principle of CD in laser ionization mass spectrometry is similar to the conventional CD, the effect is still considerably small for most UV–Vis transitions in organic molecules.

Photoelectron circular dichroism (PECD)^9,29,30^ has recently emerged as an effective gas-phase chiroptical technique with an efficiency of one to two orders of magnitude larger than CD. In PECD, the interaction between CPL and a chiral molecule results in asymmetric photoelectron angular dispersion that is identical but opposite in sign for the two enantiomers. PECD arises due to pure electric dipole transitions, therefore, it is highly efficient compared to conventional CD.

Coulomb explosion imaging is another chiroptical technique in which the momentum of each fragment is recorded when the molecule undergoes fragmentation upon rapid ionization by an intense laser pulse^31,32^. The momentum vectors of the fragments are enantio-sensitive as they are of equal magnitude but of opposite sign due to the non-symmetric behaviour of their coulomb potential. This technique is capable of imaging the absolute configuration of chiral molecules. However, it cannot be extended to complicated molecules because (1) the detection efficiency decreases exponentially for the increasing number of fragments and (2) reconstruction of the molecular structure may not be possible from the kinematic properties of the fragments.

Here we introduce a new extreme nonlinear chiroptical technique based on strong-field ionization of chiral molecules combined with mass spectrometry. Left- and right-handed elliptically polarized driving laser fields produce differential yields of doubly charged parent ion or fragment enabling chiral discrimination. The basic principle of elliptical dichroism relies on recollision physics explained by the semi-classical three-step model^33^:

1. Ionization of a chiral molecule by strong IR field in the tunneling regime defined by the Keldysh parameter $\gamma <1$^34^. For randomly orientated molecules, the laser field will preferentially ionize a subset of molecules whose molecular axes are aligned with the field.
2. Laser-induced propagation and acceleration of the ionized electron whose trajectory is influenced by the electric and magnetic field of the light; the latter corresponds to the non-dipole regime. The maximum energy of the recolliding electron is $3.17U_{p}$ where $U_{p}$ is the ponderomotive energy^35^.
3. Inelastic recollision of the liberated electron with the parent ion leading to direct ionization or excitation of the parent ion, depending on the kinetic energy of the recolliding electron.

Using limonene, $\hbox {C}_{10}\hbox {H}_{16}$, as a test case, we demonstrate Elliptical dichroism based on recollision enhanced femtosecond laser mass spectrometry (ED-REFLMS). It is an extreme nonlinear chiroptical technique with high efficiency compared to conventional CD (by at least an order of magnitude) and to PECD (by a factor of 5 for certain wavelengths). ED-REFLMS also exhibits a strong dependence on wavelength. This method is a variation of the previously examined chiral-high harmonic generation (cHHG) method^36^, where coherent radiation emitted as high order harmonics of the driving field was used to differentiate the enantiomers. However, poor conversion efficiency makes cHHG technically challenging.

## Results

Limonene consists of a cyclohexene ring with a methyl group on one end and an isopropenyl group on the other end connected to a carbon atom that serves as a chiral centre. The highest occupied molecular orbital (HOMO) is the carbon double bond (C=C) in the cyclohexene ring. $2\pi ^{-1}$ is the ground state cation of limonene with an ionization energy of 8.54 eV^37,38^. The first excited cationic state is a $1\pi ^{-1}$ molecular orbital having an ionization energy of $9.08\,\hbox { eV}$ and belongs to C=C double bond of isopropenyl group. The higher electronic excited cation states belong to the ionization of sigma bonds $\sigma ^{-1}$^30,37,39–41^. When irradiated with intense light pulses, limonene undergoes ionization and fragmentation.

### Femtosecond laser mass spectrum of limonene

**Figure 1:**
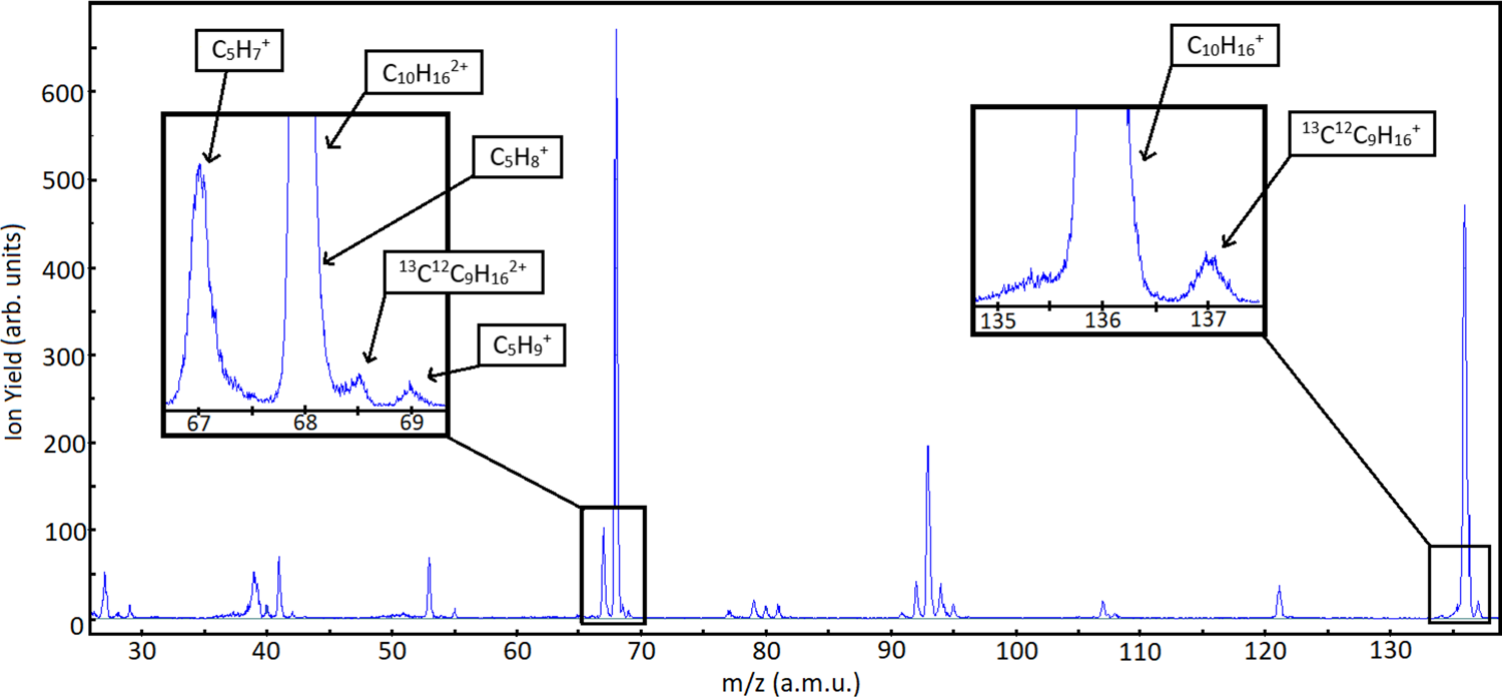
TOF mass spectrum of S-limonene at 2,000 nm and an intensity of $1.8\times 10^{14}\,\hbox { W/cm}^2$. The right inset shows magnified region of the parent ion $\hbox {C}_{10}\hbox {H}_{16}^{+}$ and its isotopomer $^{13}\hbox {C}^{12}\hbox {C}_{9}\hbox {H}_{16}^{+}$. The left inset shows the magnified region consisting of the fragment $\hbox {C}_{5}\hbox {H}_{8}^{+}$ and doubly charged parent ion at m/z = 68 along with the doubly charged isotopomer $^{13}\hbox {C}^{12}\hbox {C}_{9}\hbox {H}_{16}^{2+}$ at m/z = 68.5.

**Figure 2:**
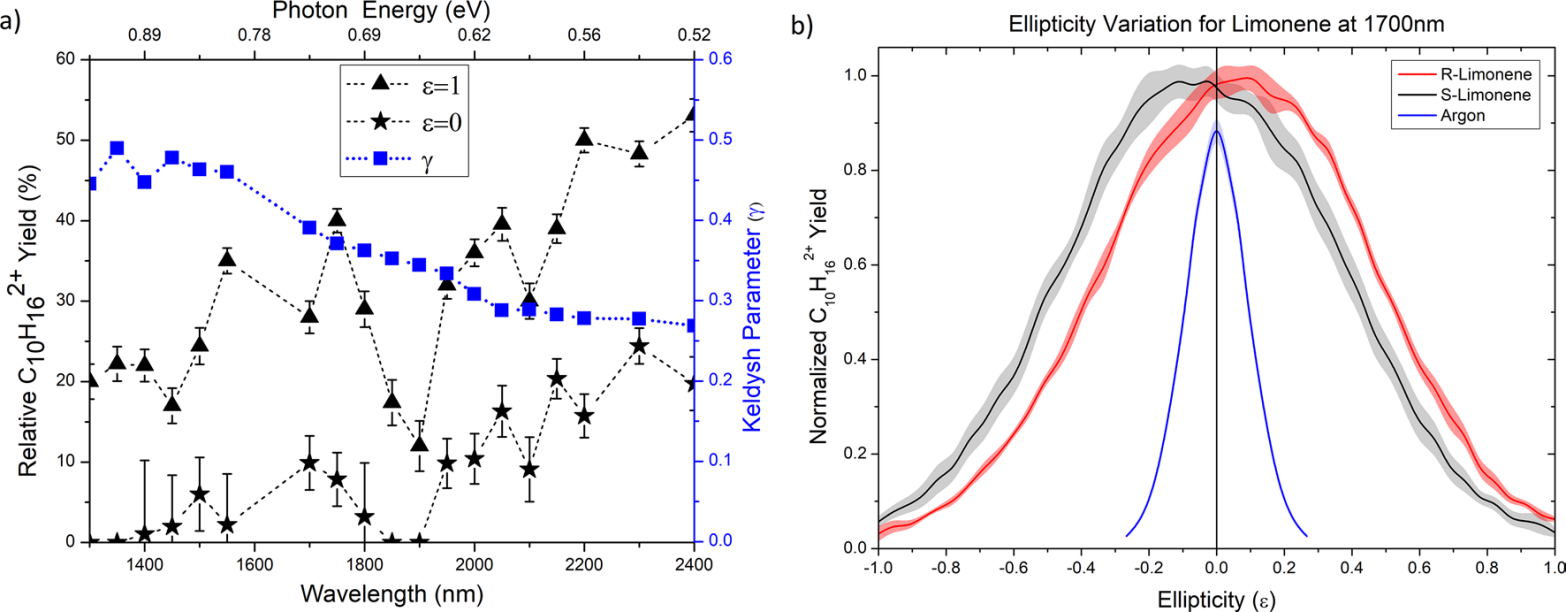
(**a**) Yield of doubly charged S-limonene relative to the fragment at m/z = 68 as a function of laser wavelength. The laser intensity was in the range of 9–$10\times 10^{13}\,\hbox { W/cm}^2$. The solid triangle represents the total double ionization yield while the solid star symbol represents contribution from sequential double ionization process. The right ordinate shows the values of the Keldysh parameter used in the experiment (blue square). (**b**) Ellipticity dependence of doubly charged limonene enantiomers, S-limonene (black) and R-limonene (red), at 1,700 nm and an intensity of $9\times 10^{13}\,\hbox {W/cm}^2$. The blue curve is the ellipticity variation for an achiral test case, argon. The shaded bands correspond to fluctuations in the ion yields.

Figure 1 shows the mass spectrum of S-limonene at 2,000 nm. The dominant peak at m/z = 68 corresponds to the fragment $\hbox {C}_{5}\hbox {H}_{8}^{+}$. However, unlike electron impact ionization, the next dominant peak corresponds to the parent ion $\hbox {C}_{10}\hbox {H}_{16}^{+}$, which exhibits a strong dependence on the wavelength. Doubly charged limonene $\hbox {C}_{10}\hbox {H}_{16}^{2+}$ overlaps with the fragment $\hbox {C}_{5}\hbox {H}_{8}^{+}$ and can be differentiated using the isotopomer of the parent ion $^{13}\hbox {C}^{12}\hbox {C}_{9}\hbox {H}_{16}^{+}$. The insets show the isotopomer of the parent ion at m/z = 137 and its doubly charged ion at m/z = 68.5. Total doubly charged limonene yield varied from 20 to 50$\%$ relative to the fragment ion signal at m/z = 68 as the wavelength increased from 1,300 to 2,400 nm, as shown in Fig. 2a. At longer wavelengths, ionization is purely in the tunneling regime ($\gamma <1$) resulting in less fragmentation. Reduced doubly charged ion yields at 1,850 and 1,900 nm is mainly due to lower yield of the parent ion. The total double ionization yield includes a contribution from both sequential and non-sequential double ionization. The latter is due to electron recollision which depends on the ellipticity, $\epsilon$, of the laser polarization and disappears for CPL. Therefore, the double ionization signal in Fig. 2a as $\epsilon$ approaches ±1 is due to sequential double ionization.

### Ellipticity variation of doubly charged ion yield

Chiral response to elliptically polarized light, which acts as a chiral reagent, is shown in Fig. 2b. Positive (negative) values of $\epsilon$ correspond to left (right) handed elliptically polarized light. The behaviour of doubly charged limonene yield, as a function of laser ellipticity, exhibits key features: (1) the yields of the two enantiomers are not maximum for linearly polarized light as expected for an atom or achiral molecule. (2) The enantiomeric response is asymmetric around the linear polarization. The double ionization signal is maximum for $\epsilon \approx \pm 0.15$. (3) R-limonene (S-limonene) yield, at 1,700 nm, is higher for positive (negative) ellipticities. (4) Chiral discrimination is not possible with linearly polarized light since the doubly charged ion yields remain the same for R- and S-limonene. Small ellipticity values are sufficient to efficiently discriminate the enantiomers.

**Figure 3:**
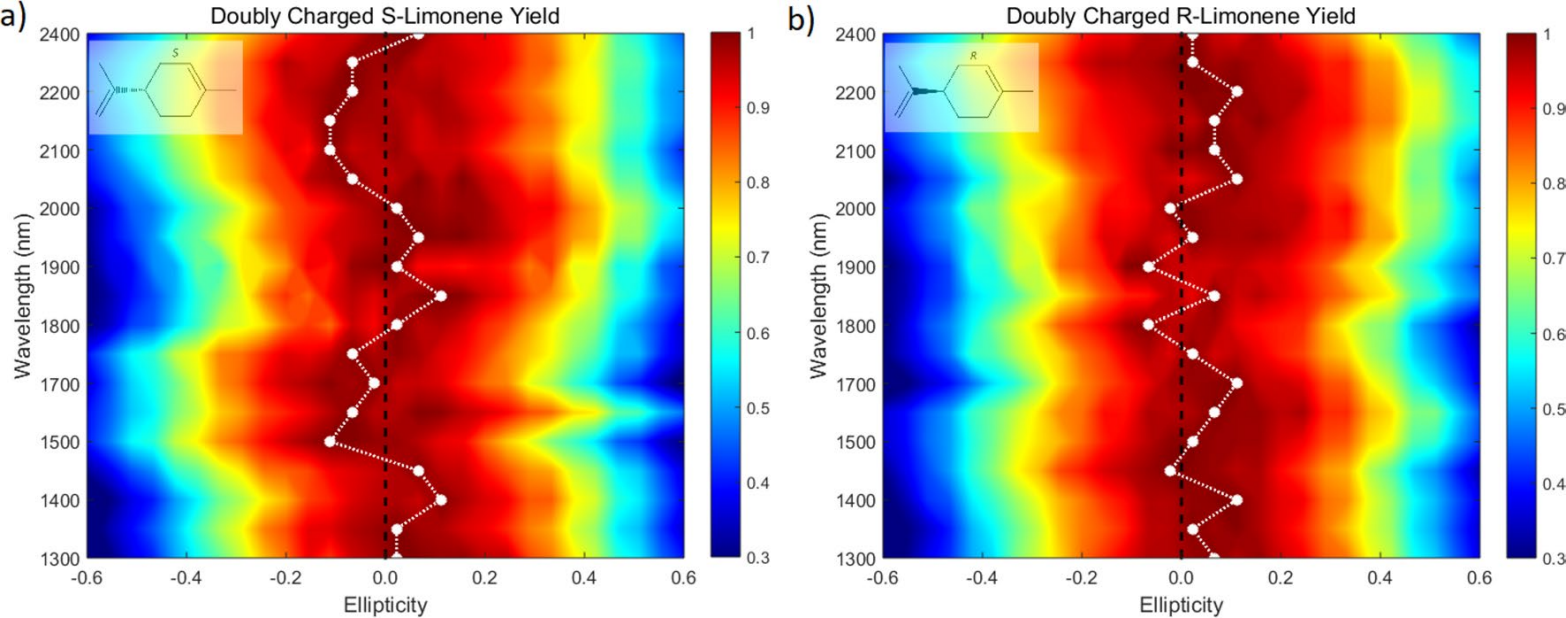
Ellipticity dependence of doubly charged S-limonene (left) and R-limonene (right) as a function of wavelength at an intensity of 9–$10\times 10^{13}\,\hbox {W/cm}^2$. The black dashed line corresponds to linear polarization and the white solid circles represent the ellipticities at which the double ionization signal is maximum.

### Wavelength effects in limonene

The colour maps of Fig. 3a,b depict the dependence of the signal intensity of doubly charged limonene as a function of ellipticities for a range of wavelengths (1,300–2,400 nm). No data could be collected at the degenerate wavelengths. The black dashed line corresponds to linear polarization and the white solid circles represent the ellipticities at which the double ionization signal intensities are maximum. The chiral response of S-limonene and R-limonene alternates about the linear polarization ($\epsilon =0$) within an ellipticity range of $-\,0.15<\epsilon <0.15$. For some wavelengths, the enantio-sensitivity is strong while for others it does not exist.

The chiral response is commonly expressed in terms of circular dichroism value. Similarly, elliptical dichroism (ED) can be defined as the ratio of the difference of the double ionization yield to the average double ionization yield of the enantiomers at a set ellipticity ($\pm \epsilon$).

1 $$\begin{aligned} ED(\pm \epsilon )=2\frac{R\left( \pm \epsilon \right) -S\left( \pm \epsilon \right) }{R\left( \pm \epsilon \right) +S\left( \pm \epsilon \right) } \end{aligned}$$

**Figure 4:**
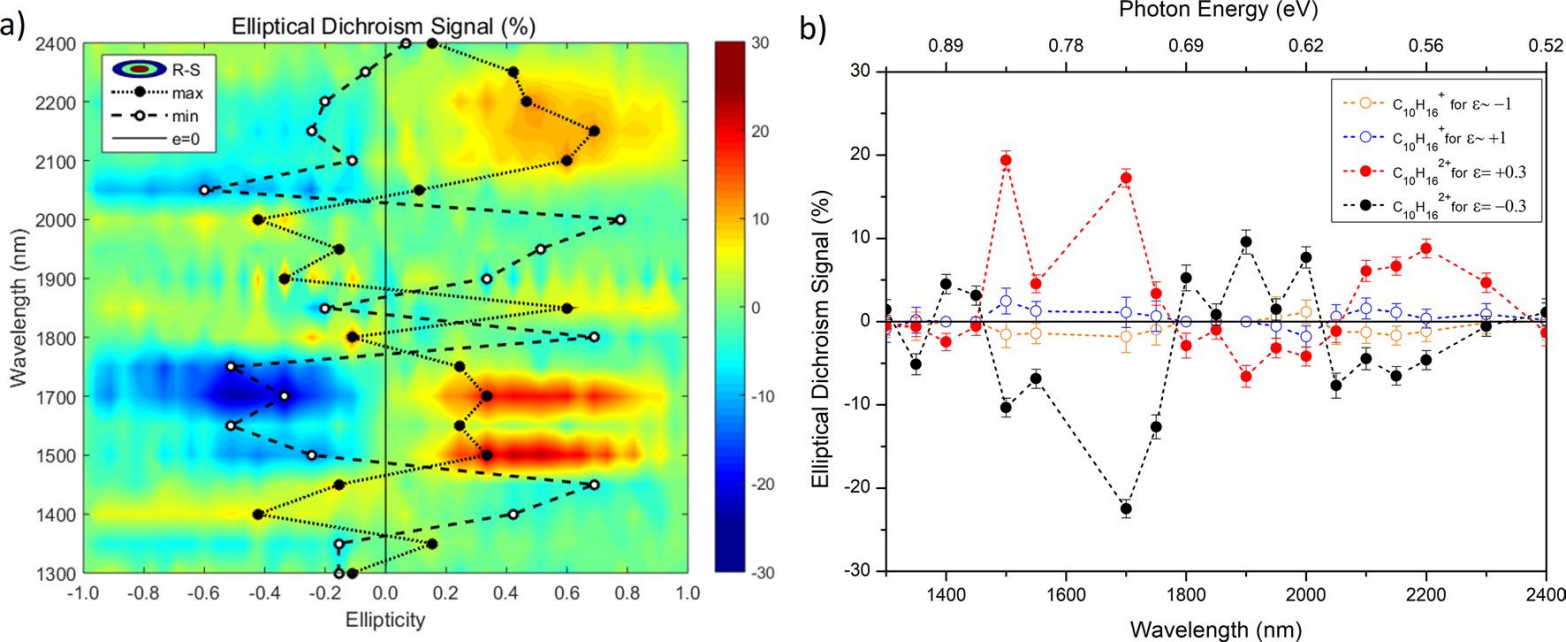
(**a**) Chiral signal represented by elliptical dichroism for $\hbox {C}_{10}\hbox {H}_{16}^{2+}$ as a function of ellipticity and at an intensity of 9–$10\times 10^{13}\,\hbox {W/cm}^2$ over a range of wavelengths. Ellipticity values at which the ED signal is minimum (maximum) is represented by white circle (black circle) and the dashed lines are to guide the eye. (**b**) Wavelength dependent ED signal for $\hbox {C}_{10}\hbox {H}_{16}^{2+}$ at $\epsilon = +0.3$ (red solid circles) and for $\epsilon = -0.3$ (black solid circles). Also, shown is the CD signal for $\hbox {C}_{10}\hbox {H}_{16}^{+}$ at $\epsilon = +1$ (blue open circles) and for $\epsilon = -1$ (orange open circles). The error bars represent the fluctuations in the ion yields and the dotted lines are to guide the eye. The laser intensity was in the range of 9–$10\times 10^{13}\,\hbox {W/cm}^2$.

Figure 4a shows the chiral signal expressed by ED for doubly charged limonene as a function of ellipticity for different wavelengths. Solid (open) circles represent the ellipticity values at which the ED signal is maximum (minimum) at a given wavelength. The chiral signal oscillating between positive and negative values indicates the double ionization yield of R-limonene being dominant for some wavelengths while S-limonene is dominant for the other wavelengths.

**Figure 5:**
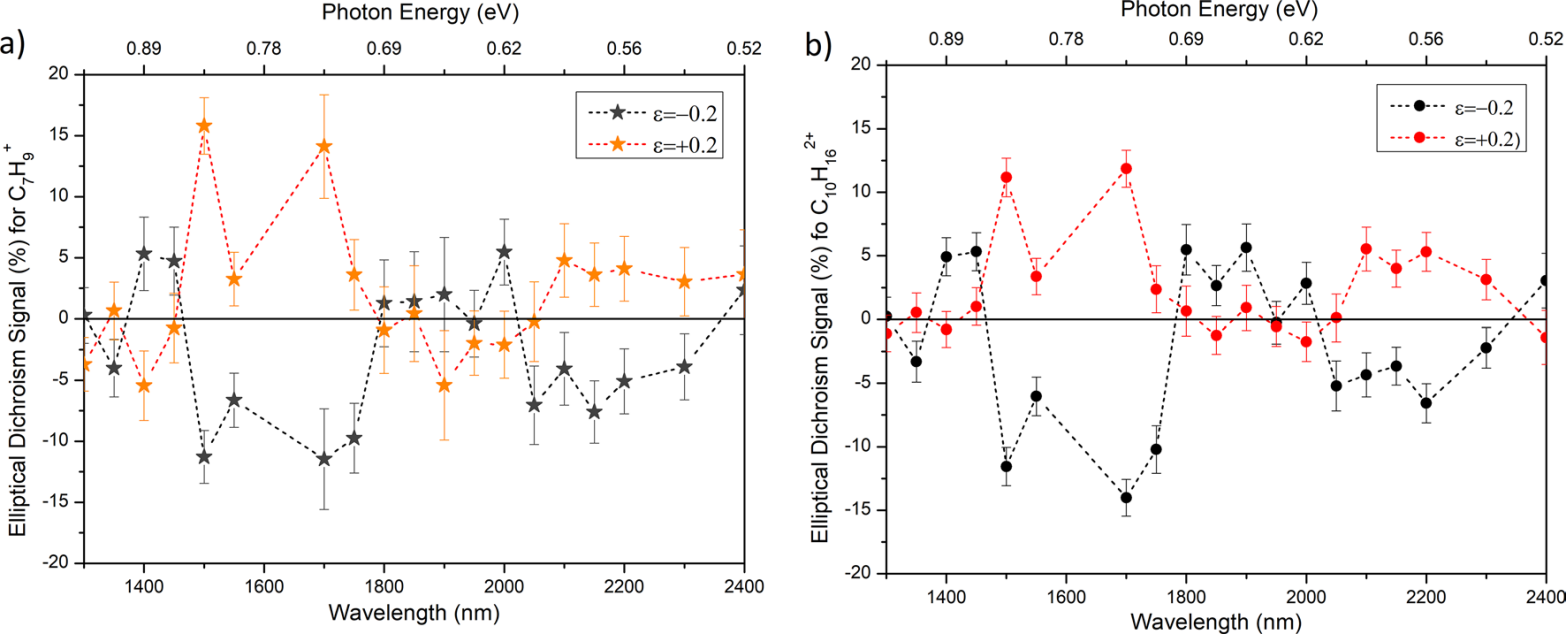
Wavelength dependence of ED signal (**a**) for singly charged limonene fragment $\hbox {C}_7\hbox {H}_9^+$, and (**b**) doubly charged limonene at $\epsilon =\pm 0.2$. The error bars represent fluctuations in the ion yields. The laser intensity was in the range of 9–$10\times 10^{13}\,\hbox { W/cm}^2$.

A full ellipticity scan is not essential to study wavelength-dependent chiral response. Instead, a wavelength scan at a fixed ellipticity should suffice, as shown in Fig. 4b for $\epsilon = 0.3$ or in Fig. 5a for $\epsilon =0.2$. Figure 4b shows the wavelength dependence of the chiral signal for singly charged limonene for CPL ($\epsilon = \pm 1$) and doubly charged limonene for $\epsilon =\pm 0.3$. The following three key features are exhibited:

1. A typical laser mass spectrometer measures the CD signal using CPL. In limonene, a CD signal of $0.16\%$^25^ was measured previously at a wavelength of 213 nm. In our measurements, in the wavelength regions of 1,500–1,750 nm and 1,950–2,300 nm, the CD signal is less than $2\%$. At other wavelengths, the singly charged ion yield was too low to measure the chiral signal. Similar results were obtained for elliptically polarized light.
2. By monitoring the doubly charged limonene yield, which arises from recollision, the chiral signal represented by ED increased to $\sim \,20\%$ at 1,700 nm with elliptically polarized light, an enhancement by up to a factor of 10. The enhancement factor peaks between $\epsilon = \pm 0.2$ and $\epsilon = \pm 0.4$ (Fig. 4a) and disappears for linear and circular polarization. As discussed later, such an enhancement of the chiral signal can arise from the recollision dynamics in the presence of the laser magnetic field. For comparison, the chiral signal measured by PECD using REMPI at 420 nm was at $4\%$^39^.
3. For some wavelengths, the positive and negative ED signals for doubly charged limonene mirror each other. At certain other wavelengths, the chiral signal is non-existent. In 1,500–1,750 nm and 2,050–2,300 nm wavelength regions, R-limonene produces more double ionization than S-limonene for positive ellipticities. Whereas, S-limonene produces higher doubly charged yield relative to R-limonene from 1,800–2,000 nm.

In molecules where it is not feasible to identify the doubly charged parent ion because it is either unstable or cannot be differentiated from a fragment, a chiral response can still be studied by monitoring recollision induced fragment. Figure 5a shows the wavelength dependence of ED signal for singly charged $\hbox {C}_7\hbox {H}_9^+$ fragment ion at an ellipticity of $\epsilon = \pm 0.2$. Chiral signal exhibits larger fluctuation due to lower yield of the fragment ion relative to the doubly charged yield. For comparison, the ED signal for doubly charged limonene at the same ellipticity value is shown in Fig. 5b. The ED signal and the wavelength dependence are nearly the same whether one monitors the fragment or doubly charged.

## Discussion

Enantio-sensitivity always requires a sign dependent response between the enantiomers. In CD, the probability of measuring an enantio-sensitive transition is proportional to the matrix element involving the electric and magnetic dipole interference term or by the molecular rotatory strength which is sign variant under improper rotations^4–6,42^. In PECD, the chiral response is described by the differential cross-section of a photoelectron defined in terms of the angular distribution function^9,29,30^. The ionized electron exhibits a forward–backwards asymmetry in the angular distribution due to momentum conservation during the photoionization process.

Chiral discrimination by ED-REFLMS technique can be understood by considering recollision dynamics beyond the dipole approximation. Strong-field ionization is often formulated within the dipole approximation, which assumes the laser wavelength is longer than the electron trajectory or the electron velocity is much smaller than the speed of light. Therefore, the magnetic effects on the electron are ignored leading to trajectories that are always in the plane of the laser polarization. However, at higher intensities, the magnetic field begins to exert a significant force on the ionized electron and its trajectory exhibits a figure 8 motion with major axis along the direction of the electric field and the minor axis along the direction of propagation^43^. The onset of the magnetic field effect occurs when the field-induced displacement along the propagation direction approaches an amplitude that is a significant fraction of an atomic unit^43,44^. This happens at higher intensities for shorter wavelengths or at lower intensities for longer wavelengths. ED-REFLMS experiments are performed on limonene at an intensity of 9–$10\times 10^{13}\,\hbox {W/cm}^{2}$ in the near IR region (1,300–2,400 nm) which falls in the non-dipole non-relativistic regime.

There are three possible mechanisms where the laser magnetic field plays a role in the recollision dynamics. All are described in terms of the time evolution of the chiral system at each step of the recollision process within the non-dipole approximation.

**Figure 6:**
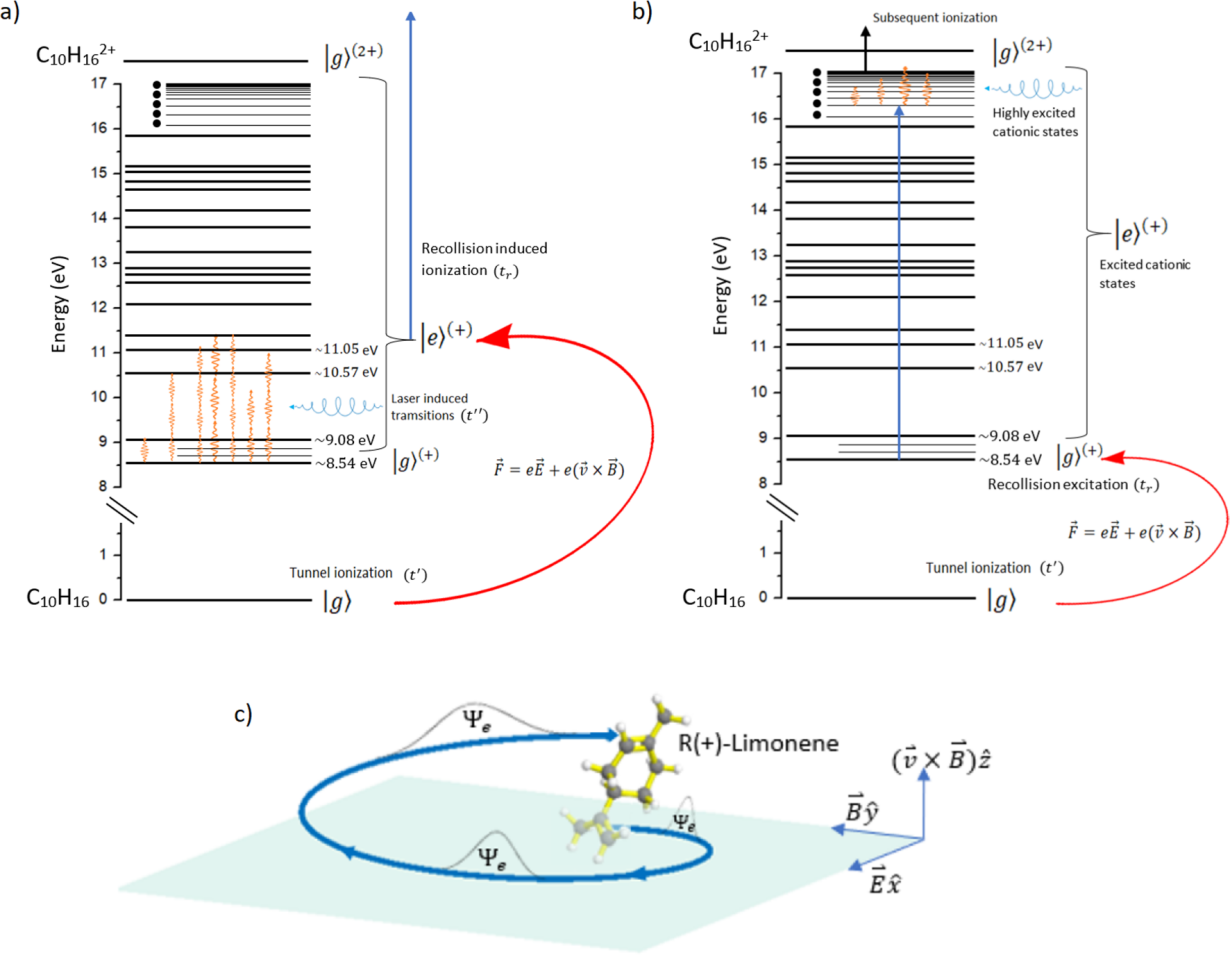
Energy level diagram illustrating chiral sensitive mechanisms 1 and 2 (**a**,**b**). The $\left| g\right\rangle$ and $\left| g\right\rangle ^{\left( +\right) }$ represent the ground state of neutral and cationic states of $\hbox {C}_{10}\hbox {H}_{16}$. (**a**) Evolution of the parent ion from $\left| g\right\rangle ^{\left( +\right) }\longrightarrow \left| e\right\rangle ^{\left( +\right) }$ cationic states induced by the elliptically polarized field (orange arrows) as the electron propagates in the continuum (red arrow). Recollision occurs on excited cationic state $\left| e\right\rangle ^{\left( +\right) }$ of the molecule and the second electron is liberated to the continuum (blue arrow) resulting in non-sequential double ionization. (**b**) Evolution of the parent ion after recollision from the $\left| g\right\rangle ^{\left( +\right) }$ to highly excited cationic states $\left| e\right\rangle ^{\left( +\right) }$ (blue arrow). The elliptically polarized laser field induces electric and magnetic transitions (orange arrows) in these highly excited states that subsequently undergo field ionization. The energy levels were obtained from limonene photoelectron spectrum^37,38^. (**c**) Schematic showing the electron trajectory in an elliptically polarized field along with asymmetric transverse momentum spread.

1. Mechanism 1: Evolution of the parent ion during electron propagation in the continuum.
  Between the ionization time ($t'$) and the recollision time ($t_{r}$), the elliptical laser field can interact with a second electron in the ground state of the cation $\hbox {C}_{10}\hbox {H}_{16}^{+}$ and induce electric dipole (E1) and magnetic dipole (M1) transitions at time $t''$. The interaction will be chiral sensitive when both the E1 (polar-vector) and M1 (pseudovector) transitions are involved. Recollision takes place on an excited state of the parent ion, as shown in Fig. 6a. For molecules containing C=C bond, a $\pi \rightarrow \pi ^{*}$ transition is E1 allowed. However, the M1 transition is forbidden in an achiral system. For a chiral system, the M1 transition is nonzero due to the asymmetrical properties of the enantiomers and is sign variant when either the ellipticity of light or the enantiomer is switched^45–47^.
  In limonene, E1–M1 transitions can promote the singly charged ion from ground state $\left| g\right\rangle ^{\left( +\right) }$ to an excited electronic cation state $\left| e\right\rangle ^{\left( +\right) }$ (orange arrows in Fig. 6a) as the ionized electron propagates in the laser field (red arrow). Recollision occurs on the excited cationic state $\left| e\right\rangle ^{\left( +\right) }$ at time $\hbox {t}_r$. Due to chiral-sensitive transitions induced by the elliptical field, electron densities in the excited cationic states are enantiomer dependent. After recollision, the double ionization signal (blue arrow) is dominant for one enantiomer compared to the other for the same ellipticity. This mechanism is similar to the “type-II” mechanism of cHHG^36^ and is also analogous to CD. However, E1–M1 transitions in CD occur in the ground state of the neutral molecule while in ED-REFLMS they occur in the parent ion.
2. Mechanism 2: Evolution of the parent ion after recollision.
  In recollision, the maximum kinetic energy for the returning electron is $3.17U_{p}$ when it is released into the continuum near the peak of the laser field. Electrons can also be released into the continuum before or after the peak, resulting in long or short trajectories. Soft recollisions can occur for electrons with lower ponderomotive energies from either shorter trajectories, or from longer trajectories that return to the parent ion at a clipping angle. Depending on the electron impact cross-section for the parent ion, the inelastic collision can promote the cation to a highly excited Rydberg-like state instead of removing the second electron (blue arrow Fig. 6b). The remnant laser field interacting with the highly excited cation can induce chiral sensitive transitions (orange arrows), and eventually lead to removal of the second electron.
3. Mechanism 3: Evolution of the liberated electron.
  In an elliptically polarized laser field, an electron in the continuum acquires a helical trajectory due to the influence of the magnetic field (Fig. 6c). Since the helicity of the electron trajectory is defined by the handedness of the light, it behaves like a chiral reagent. A chiral response can arise when (a) for a given electron helicity the collisional cross-section is different between the two enantiomers, or (b) the recolliding electron interacts with different regions of an enantiomer for opposite ellipticities.
  In addition, the tunneling electron acquires an asymmetry in its initial transverse momentum along the direction of propagation due to the Lorentz force. This asymmetry in the electron transverse momentum translates into an asymmetry in double ionization probabaility^48^. In the dipole regime, with $\mathbf {E}$-field along $\hat{x}$-axis, the initial transverse momentum of the electron is symmetric in the $\hat{y}$–$\hat{z}$ plane. The magnetic field together with recollision dynamics acts as a gate selecting only a subset of the initial tunneling-electron momenta that are opposite to the propagation direction of the laser field. Only this subset of electrons can lead to double ionization^48^. The asymmetry is either in a positive or negative $\hat{z}$-direction depending on the direction $\mathbf {B}\hat{y}$-field (handedness of incident elliptical light).
  For a chiral molecule in the presence of an elliptically polarized field, the asymmetric electron wavepacket behaves like the chiral reagent. Thus, the combined effect of breaking the symmetry of the electron momentum distribution and out of plane electron trajectories can lead to different double ionization yields of limonene enantiomers for a given ellipticity of light (Fig. 6c). This is a universal phenomenon and becomes prominent at longer wavelengths and higher intensities.

All three mechanisms can occur simultaneously in limonene. However, mechanisms 1 and 2 require magnetic dipole transitions in the cation that can still be relatively weak. Together with the universal mechanism 3, they can give rise to the observed enhancement. Any insight into such efficient extreme nonlinear chiroptical response requires new theoretical tools that take into account the non-dipole effects on strong-field ionization of molecules.

Wavelength dependence of chiral signal has been observed in chiroptical techniques such as absorption CD and PECD^37,45^. In absorption CD, it is attributed to the Cotton effect^49,50^, a change in the extinction coefficients of S- and R-limonene for right and left circularly polarized light^5,49^. In single and multi-photon PECD of limonene, varying the photon energy led to (a) modulation of the chiral signal mirroring the principal vibrational features observed in the photoelectron spectrum, and (b) flipping of the forward–backwards asymmetry in the angular emission of electrons relative to the photon beam direction^30,37,38^. The wavelength effects of the chiral signal are attributed to the excitation of vibronic states of the cation and/or intermediate electronic states of the neutral molecule.

Although CD and PECD involve excitation of intermediate states, since PECD arises from pure electric dipole interaction, the chiral signal is several orders of magnitude greater than the absorption CD. Currently, understanding the role of the intermediate resonances is subject of experimental and theoretical investigations. Recently it was demonstrated, by decoupling bound–bound and bound–continuum transitions, that the helicity of the light pulse that drives the bound–bound transitions has a negligible effect on the PECD^51,52^. However, circularly and linearly polarized laser pulses still lead to different PECDs because of the angular dependence of the excitation process, which induces different anisotropies.

In ED-REFLMS, where the photon energy was varied from 0.51 to 0.95 eV, all three mechanisms that can contribute to the chiral signal are wavelength dependent. However, mechanism 3 alone cannot give rise to the flipping of the chiral signal with laser wavelength as it does not involve any intermediate states of the cation. In mechanism 1, when an electron is promoted into the continuum, the laser field is still intense enough to induce vibronic and multiphoton electronic excitation of the cation $\hbox {C}_{10}\hbox {H}_{16}^{+}$. Some of these bound-bound multiphoton transitions can exhibit varying responses in the two enantiomers for different wavelengths. Therefore, upon recollision, the excited state dynamics influence the double ionization rates.

For example, from 1,500 to 1,750 nm where R-limonene produced more double ionization signal than S-limonene, the HOMO-2 at 10.57 eV and HOMO-3 at 11.05 eV can be accessed by 3-photon and 4-photon excitation, respectively. Similarly, from 2,150 to 2,300 nm they can be accessed 4-photon and 5-photon excitation, respectively. In the wavelength range where S-limonene produced more double ionization signal than R-limonene, HOMO-3 can be accessed by 4-photon excitation over the entire range, whereas HOMO-2 can be accessed only from 1,750 to 1,830 nm by 3-photon excitation.

In mechanism 2, the parent ion is promoted to a highly excited Rydberg-like state. At recollision time, the field is minimum, decreasing the probability of multi-photon absorption. The induced transitions are, therefore, most likely from 1-photon absorption to a dense manifold of vibrational Rydberg-like cationic states. In either of the above mechanisms, laser-induced transitions access different excited higher electronic and vibronic cationic states. The change of wavelength can affect the allowed molecular orbital transitions within an enantiomer. Small variation in the orientation of the E1 and M1 dipole transitions alters the coupling term which favours certain transition channels over others directly influencing the chiral signal detected by ED-REFLMS.

The similarities between the elliptical dichroism signal for double ionization and fragmentation can be understood by considering the complex potential energy surfaces of limonene. Upon photoionization, the molecular ions can undergo fragmentation through a multitude of dissociation pathways via intermediary states where several rearrangement reactions occur prior to dissociation^53^. The ionization energy of limonene is $\sim \,8.54\,\hbox { eV}$ while the appearance energies of fragment ions such as $\hbox {C}_9\hbox {H}_{13}^+$, $\hbox {C}_8\hbox {H}_{12}^+$, $\hbox {C}_7\hbox {H}_9^+$ and $\hbox {C}_5\hbox {H}_8^+$ are in the range of 9–11.5 eV. The pathways to these fragment ions are direct elimination of the associated neutral fragment or sequential elimination of $\hbox {CH}_{3}$ and $\hbox {C}_2\hbox {H}_4$. The same fragment ions can also arise from the dissociation of excited di-cationic states, in which case, two charged fragment ions are formed that have appearance energies close to the double ionization threshold.

In mechanism 1, recollision takes place on excited cationic states. Under normal circumstances, these excited cationic states evolve in time leading to more fragmentation. However, because the recollision dynamics occur on a short time scale of 2.5–5.5 fs (2/3 of an optical cycle for wavelengths in the range of 1,300–2,400 nm), much shorter than the dissociation time, the molecule undergoes a direct transition from excited cationic state to either ground di-cationic state (double ionization) or excited di-cationic state (fragmentation). In mechanism 2, soft recollision with the parent ion leads to excitation of Rydberg like cationic states. The remnant laser field interacting with highly excited cationic states can induce chiral sensitive transitions leading to the removal of second electron or fragmentation. Coincidence measurements can shed light on the relative influence of the two mechanisms and on the origin of the fragment ion from the dissociation of excited cationic or di-cationic states.

ED-REFLMS is a highly efficient technique where the chiral response can be obtained from either recollision-induced fragment or doubly charged ion (Fig. 5). The technique is ideal for molecules that produce stable doubly charged molecular ions which can be easily identified from fragment ions. In large molecules, the detailed molecular structure can affect ionization. So, when fragmentation overwhelms ionization, the technique can still be used to discriminate enantiomers provided the fragment ions produced by recollision are still dominant. The technique would be severely limited when contributions from sequential processes outweigh the non-sequential processes. This could be the case for some complex molecules.

In principle, ED-REFLMS does not require any ellipticity variation. The wavelength response of the chiral signal can be obtained at a fixed ellipticity (Figs. 4b, 5). This is analogous to CD spectroscopy where the differential absorption of CPL is measured for different wavelengths, albeit with better efficiency. Moreover, ED-REFLMS enables quantitative chemical analysis of chiral substances by performing mass selective spectroscopy even in a mixture of substances without prior chemical separation. In addition, extreme nonlinear chiroptical techniques like cHHG and ED-REFLMS serve as a benchmark for testing and development of theoretical tools that include non-dipole effects in strong-field ionization of molecules. Such tools enable interpretation of the fundamental relationship between chiroptical properties and detailed molecular structure.

## Methods

### Experiment

In the ED-REFLMS technique, near-infrared femtosecond laser pulses are focused onto a randomly orientated limonene molecule $\hbox {C}_{10}\hbox {H}_{16}$ in gas phase by a parabolic mirror in a deferentially pumped vacuum chamber housing a time-of-flight mass spectrometer (TOFMS). The ions produced in the interaction region of TOFMS, operating in a Wiley–McLaren geometry, are accelerated and propagated through a field-free region of 30 cm where they are detected by a micro-channel plate. The ion signal is then amplified, discriminated and recorded by a time digitizer to generate a TOF mass spectra. Mass calibration was achieved using Xenon atoms and the spectrometer has a FWHM mass resolution of 550 at an $m/z = 132$.

Both enantiomers of limonene (from Sigma Aldrich) have sufficient vapour pressure at room temperature to be introduced into the vacuum chamber in gas phase through a precision leak valve. Limonene underwent a freeze and thaw cycle to minimize contamination of the interaction region. The pressure inside the vacuum chamber was maintained at $\sim \,1.2\times 10^{-7}$ Torr. To minimize number density fluctuations in the interaction region, limonene was maintained at $25\,^{\circ }\hbox {C}$ using a temperature bath and the gas line along with the leak valve were maintained at a positive temperature gradient. S-limonene and R-limonene have a purity of $96\%$ and $97\%$, respectively.

Since limonene has low ionization potential, experiments were performed in the wavelength range of 1,300–2,400 nm to ensure adiabatic ionization. These wavelengths were obtained from an optical parametric amplifier (TOPAS-C) pumped by a Ti:sapphire laser system (Spitfire, Spectra-Physics) operating at 1 KHz repetition rate, producing $\sim$ 40 fs pulses with a maximum pulse energy of $\sim$ 2.5 mJ. The pulse duration was monitored on a shot-to-shot basis by a single-shot autocorrelator. A half-wave plate polarizer combination was used to control the pulse energy. A fast photodiode continuously monitored the laser pulse energy incident on a 3 mm thick quartz window on the vacuum chamber.

A quarter-wave plate (QWP) on a motorized rotation stage (with a $\pm \,0.005^{\circ }$ resolution) controlled the ellipticity, $\epsilon$, of the laser polarization. For each laser wavelength, the QWP was calibrated and $\epsilon =0$, corresponding to linear polarization, was accurately determined from the ellipticity dependence of doubly charged argon. The maximum ellipticity obtained was $\epsilon = \pm 0.96$ corresponding to nearly circular polarization. The uncertainty in ellipticity was $\pm \,0.02$.

Doubly charged limonene yields were recorded for each enantiomer by varying the ellipticity from $\epsilon =-1$ (RCP) to $\epsilon =1$ (LCP) in at least two different quadrants of the rotating QWP. Fluctuations of the ion yields and error bars related to the elliptical dichroism signal were computed based on repeated measurements under similar conditions. The ellipticity was varied in steps of $0.0\bar{4}$ around linear polarization and in steps of $0.0\bar{8}$ beyond $\epsilon =0.2$. ED-REFLMS measurements in limonene were performed at an intensity of 9–$10\times 10^{13}\,\hbox {W/cm}^2$ over the wavelength range of 1,300–2,400 nm, below the measured saturation intensity of $\sim \,$ 12–13 $\times 10^{13}\,\hbox {W/cm}^2$. At each wavelength, the intensity was calibrated by measuring the saturation intensity of argon.
